# Placebos Are Part of the Solution, Not the Problem. An Exemplification of the Case of Antidepressants in Pediatric Chronic Pain Conditions

**DOI:** 10.3389/fpsyt.2019.00998

**Published:** 2020-01-21

**Authors:** Cosima Locher, Jens Gaab, Charlotte Blease, Marc Inderbinen, Linda Kost, Helen Koechlin

**Affiliations:** ^1^ Division of Clinical Psychology and Psychotherapy, University of Basel, Basel, Switzerland; ^2^ Department of Anesthesiology, Critical Care and Pain Medicine, Boston Children's Hospital, Harvard Medical School, Boston, MA, United States; ^3^ School of Psychology, University of Plymouth, Plymouth, United Kingdom; ^4^ OpenNotes Keane Scholar, Beth Israel Deaconess Medical Center, Boston, MA, United States

**Keywords:** open-label placebo, antidepressants, chronic pain, children, adolescents

## Introduction

Chronic pain is highly prevalent among adolescents and up to one in four youths will develop chronic pain (1). Also, more than 10% of hospitalized children and adolescents show features of chronic pain (2), which is inherently linked to emotional distress and functional disability (3). Interventions for chronic pediatric pain comprise a range of treatment approaches, among them antidepressants (ADs). Pharmacological treatment indications for pediatric populations are usually based on data extrapolated from adults where ADs are described to be effective and frequently used for the treatment of chronic pain (4–6). Furthermore, chronic pain is multi-faced, therefore the use of ADs may be utilized not only to address pain but also underlying or comorbid psychiatric symptoms such as depression, anxiety, or different sleep disorders (4, 7). However, although ADs are frequently used in clinical practice, a recent Cochrane review could only include four studies that examined the use of ADs in pediatric chronic non-cancer pain and was therefore not able to report effect sizes (4). In addition, the small to moderate effect sizes reported in many pediatric AD trials in the common psychiatric disorders are potentially due to large placebo effects, with effect sizes as high as high as 1.57 (Hedges *g*) (8). Moreover, the use of ADs in children and adolescents remains controversial (9), as severe adverse events—including an increased risk of suicidal thoughts and behaviors–have been reported, leading to a black box warning by the Food and Drug Administration (FDA) in 2004 (10).

### Placebo Effects and Mechanisms in Children and Adolescents

To date, placebo effects in children and adolescents with clinical conditions are poorly understood. A review from 2013 concludes that from all citations found with the search term “placebo,” only around 2.5% discussed the placebo effect in children and adolescents, and most of them were conducted in the field of attention-deficit hyperactivity disorder (ADHD), depression and migraine (11). Existing literature reports generally higher placebo effects in pediatric populations compared to adults across different disorders (e.g., obsessive compulsive disorder, anxiety disorders, depression, and epilepsy) (11–14). For children with ADHD, Waschbusch et al. (15) reported average rates of positive response to placebo to be up to 20–30% in a review of drug treatments. Notably, while this is an interesting finding, it is not equivalent to a placebo effect which requires a no-treatment control group for comparison (16).

It has been proposed that some of the psychological mechanisms underlying the placebo effect in children and adolescents are similar to those in adults, such as the patient-provider relationship, positive expectations (of parents and child), a plausible narrative, as well as a powerful ritual (11, 17–19), but the specific underlying mechanisms of placebo effects in children and adolescents remain poorly understood. It has, for example, been argued that placebo analgesia can be induced *via* social observational learning (20); children and adolescents may be more susceptible for social learning mechanisms by imitation and role models (11). Furthermore, it has been speculated that children aged 6 years or older have higher learning capacities than adults, at least for the process of associative learning. Associative learning, in turn, is a well-known underlying mechanism associated with placebo responses (11).

### Open-Label Placebos—A Promising Approach in Adults as Well as in Children and Adolescents

Given the often large and clinically significant effects of placebos, an important question is how these effects can be harnessed in clinical practice within an ethical framework and without the use of deception (21, 22). Here, the idea of open-label placebo (OLP) treatment, i.e., full disclosure of the placebo being nothing but a placebo, might seem counterintuitive to the common perception of placebos, but has received increased scientific interest in the last decade. Overall, in adults, there is evidence for the clinical potential of OLPs for several pain conditions such as irritable bowel syndrome (23), migraine (24), chronic low back pain (25), and experimental heat pain (26, 27). A meta-analysis by Charlesworth and colleagues (28) summarized the effects in pain and non-pain conditions: patients in the OLP group revealed significantly greater clinical improvement than those in the control groups (standardized mean difference = .88). OLP studies typically employ an expectancy paradigm, where OLP are provided with a scientific rationale: a positive albeit realistic expectancy is verbally fostered with the use of the four discussion points explaining that a) placebos are effective, b) classical conditioning is a possible mechanism, c) compliance is important for outcome, and d) positive expectations increase placebo effects, but are not necessary (23, 29). It has been proposed that the effects of OLP are unlikely to be limited to the administered placebo, but also rely on the patient-provider interaction and the treatment rationale (30).

To date, only two studies have directly tested the possibility of harnessing OLP in children and adolescents (31, 32). In a pilot study (32), Sandler and colleagues examined whether OLPs can help for the medication dose reduction in children with ADHD for the first time. Patients were randomly assigned to one of two orders of experimental conditions. Across the sample as a whole, they found that the ADHD symptoms remained the same when the dose of stimulant medication was reduced with the help of OLP but deteriorated when the dose was reduced without OLP. In their follow-up study (31), the authors randomized children with ADHD to a medication dose reduction with OLP, a medication dose reduction without OLP, and a control group (i.e., the “no reduction condition”) after having been on a stable and established regimen of amphetamine salts. They found that compared to the group that received the full medication dose over the whole study period, the medication dose reduction with OLP group had similar severity ratings of parent-reported ADHD symptoms. Furthermore, treatment emergent side effects were lowest in the medication dose reduction with OLP group (31). This suggests that a reduction of 50% of the medication dose, replaced by an OLP, leads to a comparable change in symptom severity as does the full dose of the medication while experiencing only a minimal amount of side effects.

### How to Harness the Placebo Effect in Children and Adolescents

Based on these preliminary findings, we suggest that OLP could inform a promising treatment approach in children and adolescents. In order to outline our arguments, we will use the example of children and adolescents suffering from chronic pain conditions. On the one hand, OLPs could be administered as a treatment extension to the use of ADs. Here, the study design from Sandler and colleagues (31, 32) could serve as a starting point. OLPs could support children and adolescents with chronic pain to reduce the dosage of ADs by describing them as a “dose extender.” It can be hypothesized that the additional use of OLPs would not only help to avoid an increase in pain symptoms, but would also decrease the amount of side effects due to ADs. On the other hand, OLPs could be applied as a treatment alternative to the use of ADs. In this case, OLPs could either replace ADs completely (i.e., children and their parents aim for the total withdrawal of ADs) or could be the primary choice of treatment from the beginning (i.e., children and their parents aim to use OLPs instead of ADs in the first instance). In both cases, the patient-provider interaction as well as the treatment rationale play a crucial role in the administration of OLP. In the following, prime targets for this approach are identified and exemplified.

In order to transfer OLP to the field of children and adolescents, the patient-provider interaction and the rationale for the OLP should best be adapted for and targeted to this age group. *Patient-provider interaction* in this case involves not only two people, but rather a family system in which the members (i.e., in most cases one or both parents and the child) have different needs and levels of understanding. For example, Whalley and Hyland (33) examined the so-called “placebo by proxy” effect, i.e., the phenomenon that a patient's response to an intervention is affected also by the behavior of other people. They found that children's response to treatment for anger tantrums is strongly associated with the expectations, beliefs and mood of the parents. It has been argued that parental views can be in conflict with physicians' reports and their expectations can, in turn, interfere with the measured outcomes in a trial with children and adolescents (34).

Furthermore, the *treatment rationale* should be plausible and credible for both the children and their parents. Most importantly, this calls for age-appropriate explanations of the examination process and treatment delivery. Depending on the age of the patient, it may be useful to use metaphors, analogies, talk about other children's experience with the same intervention, and emphasize the potential of the intervention to help the children help themselves (32, 35). Furthermore, the treatment rationale should be adapted to the specific needs of children and adolescents suffering from pain conditions. In an experimental heat pain study, for example, children and adolescents were randomized to an analgesia-expectation or a control-expectation condition. In the former condition, the authors made use of an analogy, by telling the participants a story about a child who uses the lotion that will be tested in the experiment (i.e., a child intends to go treasure hunting in the desert and is accompanied by his/her friend, a lion. In order to be protected from the heat of the sand, the child applies a lotion to his/her hands and feet) (14). Although heat pain threshold and tolerance increased in both conditions, the predictor “magical thinking” influenced the pain perception of the pediatric population.

However, the aforementioned discussion points for OLP (23) have not yet been tested or revised for children and adolescents. We suggest that future controlled trials examining OLPs in children and adolescents with chronic pain should *a*) test and validate the OLP rationale; *b*) adapt the OLP rationale to the specific age group in order to ensure the credibility and plausibility of the treatment approach. Ideally *c*), the rationale should include disease-specific elements to clarify which symptoms may be influenced in particular ways by the OLP. Importantly *d*), parents would have to be furnished with understandable and up-to-date evidence. Finally *e*), the involvement of the parents would support a good patient-provider interaction, acknowledging the different members of the family system.

### Medical-Decision Making and Informed Consent

As the administration of OLPs is explicitly transparent, it is especially important that patients are provided with adequate information to reach a decision about using this treatment. For OLP studies in adults, it has been argued that some patients might not understand the information given, that the time in the consultation is limited and, conclusively, that it is hardly possible for physicians to provide exhaustive details about the intervention (36). This is specifically accentuated in children and adolescents, where developmental aspects critically influence the decision-making capacity. In the context of medicine, four components of decision-making have been found to be key: the ability to express a choice (e.g., being able to communicate); the ability to understand the information provided (e.g., sufficient intelligence and language proficiency, attention, and memory); the ability to reason (e.g., weighing risks and benefits, consider consequences); and the ability to appreciate the relevance of the situation (e.g., abstract thinking, theory of mind) (37). In general, it has been argued that children around the age of 12 are sufficiently decision-making competent (32, 33), while as younger children are not considered competent enough to act for themselves (38). Considering that informed consent in pediatrics typically involves the patient, a parent or guardian, and the clinician, we suggest that future controlled trials examining OLPs in children and adolescents with chronic pain should *a*) involve parents in treatment decisions related to the administration of OLPs. However, we recommend that *b*) the level of involvement should depend on the child's age.

### Ethical Considerations

We argue that ethical considerations are closely linked to the clinical success of OLPs. Whereas we were able to provide some suggestions for future clinical studies, we claim that it is too early to implement OLPs into clinical practice for children and adolescents suffering from chronic pain syndromes. We highlight three important considerations. First, in light of the hypothesis that placebo effects in children are partly harnessed by proxy (33), ensuring that parents have an adequate, evidence-based understanding of placebo effects and OLPs is crucial. This includes their treatment limitations, as overestimating the role of placebo effects would not only constitute an unacceptable deception, but there is also evidence that it would be counterproductive to the success of the treatment (39).

Second, and related, patients suffering from chronic pain are an especially vulnerable medical population—some of whom may have already been harmed from previous medical encounters. We suggest that the use of ADs with this population already suggests a form “psychologization” of symptoms that may be deeply troubling to patients, with the attendant notion that their conditions is “all in their head” (40). In light of this, we suggest that it will be crucial to explore the acceptability of OLPs with this patient group, for example by the use of extensive qualitative research among minors and their parents to determine opinions and attitudes about OLPs, including their understanding of how they work. Here again, ongoing assessment of patients' and parents' views will be not just be ethically important, it will likely be empirically relevant to the effectiveness of the treatment. To illustrate: if patients or their parents self-stigmatize or blame themselves for OLPs not working, this may not only be morally damaging, it may engender serious negative effects of the treatment.

Third, and related, there are likely serious deficits in placebo literacy among clinicians, including when it comes to ethical clinical use of placebos (41, 42). Before they can reasonably be expected to use OLPs, clinicians will require augmented education about these novel treatments.

## Discussion

In the treatment of chronic pain conditions in children and adolescents, the use of OLP or its underlying processes could have several potential advantages: First of all, OLPs may help to mitigate the risks of serious short- and long-term side effects associated with AD use. Second, OLP mechanisms (i.e., pill, the patient-provider interaction, and the treatment rationale) can easily be embedded in the clinical encounter and the medical model. Third, in any clinical encounter, physicians should be open about their own expectations regarding the treatment and ask about those of the patient (43). However, considering the vulnerabilities among this patient demographic as well as the lack of empirical evidence, the effectiveness and feasibility of this approach clearly needs to be evaluated in physicians, patients, and their parents.

To conclude, using OLP as an alternative or extension treatment with ADs is a promising idea, both for research and clinical practice, but it is too early to draw definitive conclusions, and we strongly argue against recommending OLPs until the potential of this treatment is better understood. Results from research in adult populations cannot and should not be transferred directly to pediatric populations. This holds true for results in AD and OLP research, and is especially important in the context of medical decision-making and informed consent. Hence, despite challenges posed by the enrollment of children in clinical trials (44), future research should make an effort to study OLP in children and adolescents with chronic pain conditions in order to do justice to developmental features that are specific to this age group and to further enhance a potentially powerful intervention for this vulnerable population.

## Author Contributions

CL and HK are responsible for the concept of the opinion paper. CL, JG, CB, MI, LK, and HK wrote and reviewed the manuscript.

## Funding

CL is sponsored by the Swiss National Science Foundation fellowship (P400PS_180730), the Swiss National Science Foundation fellowship (P400PS_186658) sponsors HK. CB was supported by a Keane Scholarship.

## Conflict of Interest

The authors declare that the research was conducted in the absence of any commercial or financial relationships that could be construed as a potential conflict of interest.
